# Upconversion Nanoparticles-Based Fluorescence Immunoassay for the Sensitive Detection of 2-Amino-3-methylimidazo [4,5-f] Quinoline (IQ) in Heat Processed Meat

**DOI:** 10.3390/s22010008

**Published:** 2021-12-21

**Authors:** Xufang Huang, Wei Sheng, Haonan Chen, Biao Zhang, Na Huang, Shuo Wang

**Affiliations:** 1 State Key Laboratory of Food Nutrition and Safety, College of Food Science and Engineering, Tianjin University of Science and Technology, Tianjin 300457, China; huangxufang2021@163.com (X.H.); chenhaonan_1997@126.com (H.C.); zhangbiao9129@163.com (B.Z.); Ninahnn@163.com (N.H.); 2 Tianjin Key Laboratory of Food Science and Health, School of Medicine, Nankai University, Tianjin 300071, China

**Keywords:** 2-amino-3-methylimidazo [4,5-f] quinoline, upconversion nanoparticles, magnetic separation, fluorescence immunoassay, heat processed meat

## Abstract

A competitive fluorescence immunoassay for the quantitative detection of 2-amino-3-methylimidazo [4,5-f] quinoline (IQ) in pan-fried meat patties was developed, using magnetic nanoparticles coupled with coating antigen as the capture probe and anti-IQ antibody coupled with NaYF_4_: Yb, Er upconversion nanoparticles as the signal probe. Under optimal conditionals, the wide detection range for IQ in phosphate buffer saline is from 0.01 to 100 μg·L^−1^ (R^2^ = 0.991) with a detection limit of 0.007 μg·L^−1^. This proposed method has been applied to detect IQ in two different types of pan-fried meat patties at varying frying times, and the IQ content in chicken patties and fish patties are 2.11–3.47 μg·kg^−1^ and 1.35–2.85 μg·kg^−1^, respectively. These results are consistent with that of the ultraperformance liquid chromatography-tandem mass spectrometry. In summary, this method can serve as a sensitive and specific test tool for the determination of IQ in processed meat.

## 1. Introduction

**Figure 1:**
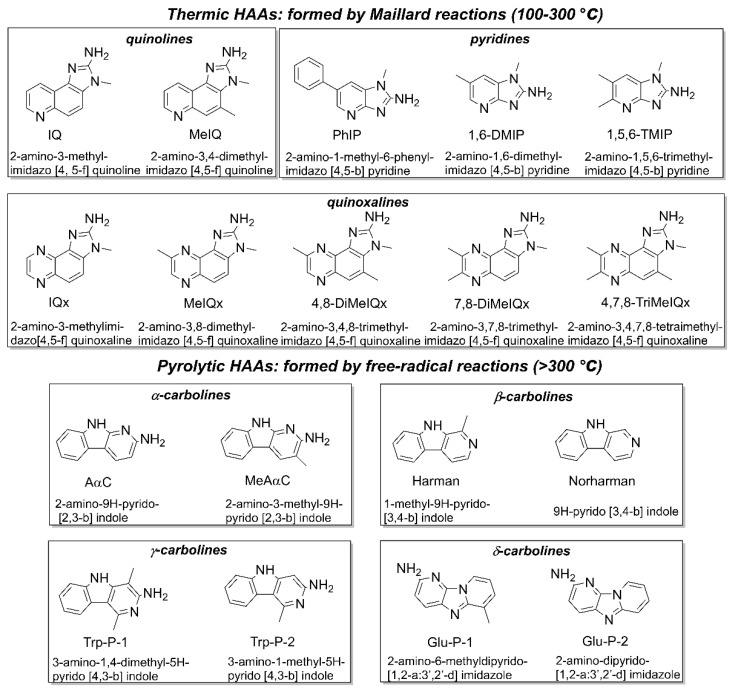
Names and molecular structures of common heterocyclic aromatic amines (HAAs).

Heterocyclic aromatic amines (HAAs) are a class of carcinogenic compounds, which form during the high-temperature cooking of protein-rich foods [1]. On the basis of the required temperature for formation, we can divide HAAs into two categories—thermic HAAs or polar HAAs (including quinoline type, pyridine type, and quinoxaline type) and pyrolytic HAAs or nonpolar HAAs (including α, β, γ, δ-carboline type). Thermic HAAs form between 100 °C and 300 °C through the Maillard-type reactions, and pyrolytic HAAs usually produce above 300 °C by direct pyrolysis of amino acids and proteins [2,3]. The names and molecular structures of common HAAs are displayed in Figure 1.

Among them, 2-amino-3-methylimidazo [4,5-f] quinoline (IQ) shows stronger mutagenicity than benzopyrene and aflatoxin B1 in the Ames *Salmonella typhimurium* test [4]. The tumor-inducing effect of IQ is also exhibited by many animal trials, including mice and non-human primates [5,6]. In 1993, based on animal trials, the International Agency for Research on Cancer (IARC) had classified several types of HAAs as possible carcinogens (class 2B), while IQ as a probable carcinogen (class 2A) for humans [7]. Given the strong mutagenicity and potential carcinogenicity of IQ, many studies have been carried out to investigate its formation mechanism and quantitative analysis. Although the exact mechanisms and processes of IQ formation were not yet completely understood, a possible pathway using creatinine and Maillard reaction products (glucose and amino acids) as precursors had been proposed [8]. Moreover, several studies have indicated that the content of IQ formed in food is primarily influenced by time, temperature, cooking methods and the precursors in raw materials [9,10].

A sensitive and accurate analytical method is necessary to study the precise formation mechanism of IQ or to further guide the meat processing method for guaranteeing food safety. Many extraction and purification methods have been proposed for IQ detection in various food matrices (including solid-phase extraction, solid-phase microextraction, dispersive liquid–liquid microextraction [11,12,13]. Several instrumental analysis methods, including high-performance liquid chromatography with different kinds of detectors (electrochemistry, ultraviolet, fluorescence, and mass spectrometry) [14,15,16,17], gas chromatography [18], gas chromatography-mass spectrometry [19], and capillary electrophoresis [20] have been constructed for IQ detection. Although the above methods are sensitive and accurate, they require complicated sample preparation and expensive apparatus. Compared with these methods, immunoassay has some merits of high specificity, easy operation, and economy. For the immunoassay to detect IQ, only two enzyme-linked immunosorbent assays (ELISAs) have been previously reported by our laboratory until now; one is based on a specific anti-IQ-antibody and the other is based on a broad-spectrum antibody, but the sensitivity of the specific assay needs to be further improved [21,22].

With the advance of nanotechnology, several nanomaterials related to signal amplification strategies have been extended into analytical methods to enhance their efficiency and sensitivity [23]. Fluorescence immunoassay, which is based on the specific binding of antigens and antibodies and the signal amplification advantages of nanomaterials, has been widely used in the analysis area. Compared with traditional down-conversion fluorescence materials, upconversion nanoparticles (UCNPs) have attracted enormous scientific interest owing to their excellent features, including large anti-Stokes shifts, biocompatibility, low autofluorescence background [24]. In recent years, UCNPs have been widely used in biological imaging, labeling, photodynamic therapy, and anti-counterfeit [25,26,27,28]. Therefore, an attempt has been made to establish a fluorescence immunoassay by combining the signal amplification effect of UCNPs and the rapid separation ability of magnetic nanoparticles (MNPs) to achieve rapid and sensitive detection of IQ in heat processed meat.

## 2. Materials and Methods

### 2.1. Materials and Apparatus

The 2-amino-3-methylimidazo [4,5-f] quinoline (IQ) and other HAAs were purchased from Toronto Research Chemicals Inc (Toronto, Ontario, Canada) and 1-(3-dimethylaminopropyl)-3-ethylcarbodiimide (EDC), C_6_H_9_O_6_Y·xH_2_O (Mw: 266.04 g·mol^−1^), C_6_H_9_O_6_Er·xH_2_O (Mw: 344.39 g·mol^−1^), C_6_H_9_O_6_Yb·4H_2_O (Mw: 422.23 g·mol^−1^), and N-hydroxy succinimide (NHS) were purchased from Sigma-Aldrich (St. Louis, MO, USA). N, N-dimethylformamide (DMF), oleic acid (OA), diethylene glycol, and 1-octadecene were purchased from Aladdin (Shanghai, China). Magnetic nanoparticles (MNPs) were purchased from Baseline (Tianjin, China). Anti-IQ polyclonal antibody was produced in our laboratory. 2-Morpholinoethanesulfonic acid (MES), polyacrylic acid (PAA), ovalbumin (OVA), 2-[4-(2-hydroxyethyl) piperazin-1-yl] ethanesulfonic acid (HEPES), bovine serum albumin (BSA), and other chemical reagents used in this study were purchased from Reagent Co., Ltd. (Beijing, China).

The morphology and size of UCNPs were examined by transmission electron microscopy (TEM) (JEOL Ltd., Tokyo, Japan). The functional groups of UCNPs were investigated by Fourier transform infrared spectrometer (Bruker, Berlin, Germany). The fluorescence intensity of UCNPs was determined by an F-2500 fluorescence spectrophotometer (Hitachi, Tokyo, Japan) equipped with an external 980 nm laser (Beijing Hi-Tech Optoelectronic Co., Ltd., Beijing, China) instead of the internal excitation source.

### 2.2. Synthesis of NaYF_4_: Yb, Er UCNPs

The synthesis method of the oleic acid-coated hydrophobic UCNPs (OA-UCNPs) was mainly based on the thermal decomposition method [29], with some modification. Briefly, 207.5 mg of C_6_H_9_O_6_Y·xH_2_O, 6.9 mg of C_6_H_9_O_6_Er·xH_2_O, 84.5 mg of C_6_H_9_O_6_Yb·4H_2_O, 17 mL of 1-octadecene, and 6 mL of oleic acid were added into a three-neck round bottom flask, and the mixture was stirred evenly. Next, the above mixture was degassed by a vacuum pump, and heated to 100 °C and kept for 20 min to remove the bound water. Then the above mixture was heated to 160 °C under argon protection for a further 30 min, followed by cooling to room temperature. Subsequently, 148.0 mg of NH_4_F and 100.0 mg of NaOH were dissolved in 10 mL methanol, and the mixed solution was added into the above flask and was stirred for 30 min. Then, the solution was heated to 80 °C and kept for 1 h to remove the methanol. Thereafter, the resulting mixture was degassed by a vacuum pump at 100 °C for 20 min and was then heated to 300 °C for a further 1 h under argon protection. After cooling, the product was centrifuged for 10 min (10,000 rpm, 25 °C), washed three times with ethanol, dried at 37 °C for 24 h, and the oleic acid-coated hydrophobic UCNPs (OA-UCNPs) were obtained.

### 2.3. Modification NaYF_4_: Yb, Er UCNPs with Polyacrylic Acid

The hydrophobic oleic acid groups on the surface of the OA-UCNPs were replaced by the hydrophilic polyacrylic acid (PAA) groups to get polyacrylic acid-coated UCNPs (PAA-UCNPs) [30]. Briefly, 60 mL of diethylene glycol and 3.0 g of PAA were added into a three-neck round bottom flask, then heated to 110 °C with vigorous stirring for 1 h under argon protection. Next, 180.0 mg of OA-UCNPs were dispersed in 12 mL of toluene by ultrasonication, and the mixed solution was added into the above flask. Thereafter, the above solution was heated to 110 °C and kept for 1 h, then increased to 240 °C and held for an additional 1 h. After naturally cooling down, the modified UCNPs were centrifuged for 10 min (10,000 rpm, 25 °C), washed three times with ultrapure water, and then dried at 37 °C for 24 h to get hydrophilic polyacrylic acid-coated UCNPs (PAA-UCNPs).

### 2.4. Preparation of Signal Probe

The signal probe was synthesized through the classical active ester method. First, 5 mg of PAA-UCNPs, 1.5 mg of EDC, and 2.4 mg of NHS were dissolved in 2 mL MES buffer (pH 5.5, 10 mmol·L^−1^) by sonication in a 10 mL flask. Then, the reaction mixture was stirred at 30 °C in a water bath for 3 h to activate the carboxyl groups on the surface of PAA-UCNPs. The above solution was then washed three times with HEPES buffer (pH 7.2, 10 mmol·L^−1^), followed by centrifugation for 10 min (5000 rpm, 4 °C). Subsequently, the above activated PAA-UCNPs, anti-IQ antibody solution, and 1.5 mL of HEPES buffer were added into a 10 mL flask. Then the resulting mixture was stirred slowly for 3 h at 4 °C. Thereafter, 15 mg of BSA was added and reacted for 1 h to block the uncombined sites on the surface of PAA-UCNPs. Then, the resulting solution was centrifuged for 10 min (5000 rpm, 4 °C) and washed three times with HEPES buffer. Finally, the obtained precipitate was redispersed into 2 mL of HEPES buffer to acquire the signal probe.

### 2.5. Preparation of Coating Antigen

The method of preparing the coating antigen refers to our previous study [21]. 1H-pyrrolo [2,3-f] quinoline (PQ), a structural analogue of IQ, was connected with two-carbon arm to obtain the hapten PQ acid (PQA). Coating antigen (PQA-OVA) was synthesized by the active ester method, and the details are as follows: first, 3.5 mg of PQA was dissolved in 200 μL DMF, then 3.5 mg of NHS and 5.8 mg of EDC were added into the above mixture in a flask. Next, the flask was incubated at room temperature and shielded from light overnight to get the activated PQA solution. Subsequently, 13.0 mg of OVA was dissolved in 1.3 mL PBS (0.01 mol·L^−1^, pH 7.4) in a 10 mL flask. Thereafter, the activated PQA solution was added into the OVA solution drop by drop and stirred at 4 °C for 12 h. Finally, the above solution was dialyzed in PBS for three days to obtain the coating antigen.

### 2.6. Preparation of Capture Probe

The active ester method was used for the synthesis of the capture probe. First, 5 mg of MNPs, 5 mg of EDC, and 4 mg of NHS were dissolved in 2 mL MES buffer in a 5 mL tube. Then the mixture was incubated on a horizontal reciprocating shaker for 1 h at room temperature. After incubation, the precipitation was separated with a magnet and washed three times with phosphate buffer saline (PBS, 0.01 mol·L^−1^, pH 7.4). Subsequently, the above activated MNPs were redispersed in 1 mL PBS buffer in a 5 mL centrifuge tube. Followed by adding the coating antigen solution into the above centrifuge tube, then the mixture was placed on the horizontal reciprocating shaker for 3 h at room temperature. Next, 15 mg of BSA was added and reacted for 1 h to block the uncombined sites on the surface of MNPs. Thereafter, the coupled compound was collected with a magnet and washed three times with PBS buffer. Finally, the obtained precipitate was resuspended with 2 mL PBS buffer to get the capture probe.

### 2.7. Test Principle and Procedure of Assay

**Figure 2:**
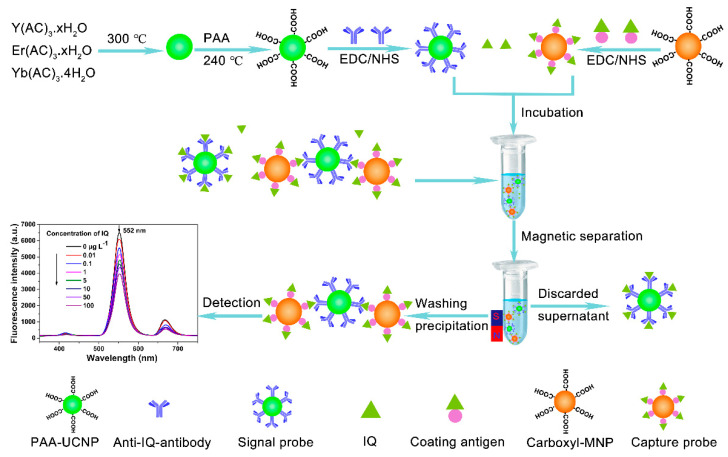
Preparation of signal probe and principle of fluorescence immunoassay.

Figure 2 shows the schematic of the fluorescence immunoassay. With the absence of IQ, the signal probe all binds to the capture probe, and a high fluorescence signal is detected. While with the presence of IQ, the target analyte and capture probe will competitively combine with the signal probe, and a low fluorescence signal is obtained. The content of IQ is obtained by recording the change in fluorescence values. Briefly, 25 µL of capture probe, 50 µL of IQ standard or sample solution, 25 µL of signal probe, and 300 µL of PBS buffer were added into a 2 mL centrifuge tube. Then, the mixture was incubated for 50 min at room temperature on the horizontal reciprocating shaker. Subsequently, the immune complex was separated by a magnet and washed three times with PBS buffer. Finally, the immune complex was resuspended into 400 μL of PBS buffer, and all fluorescence measurements were recorded by an F-2500 fluorescence spectrophotometer equipped with an external 980 nm laser.

### 2.8. UPLC-MS/MS Analysis

UPLC-MS/MS analysis was performed according to the Chinese National Standard (GB 5009.243-2016) [31] using a UPLC H-Class/Q-Trap 5500 (Waters, AB Sciex, Milford, MA, USA). A Waters CORTECS @ T3 2.7 μm 2.1 × 100 mm column was used. The mobile phase A was 15 mmol L^−1^ ammonium formate solution (pH 3.5, adjusted by formic acid) and mobile phase B was acetonitrile. The flow rate was 0.2 mL·min^−1^, the column temperature was 25 °C, and the injection volume was 5 µL. The gradient elution program was: 0 min: 95% A, 0.5 min: 95% A, 3 min: 70% A, 6 min: 40% A, 6.1 min: 5.0% A, 6.5 min: 5.0% A, 6.6 min: 95% A, 10 min: 95% A. The mass spectrometry detection was carried out in the electrospray ionization positive ion mode (ESI+) and multiple reaction monitoring (MRM) mode with the following: capillary voltage 3.0 kV, source block temperature 100 °C, desolvation temperature 350 °C, desolvation gas 800 L·h^−1^, cone gas 50 L·h^−1^, cone voltage 40 V. The precursor ion (m/z) was 199.2, product ions (m/z) were 184.2 and 157.2, and the collision energies were 25 (V) and 35 (V), and the qualitative ion (m/z) was 184.2. IQ was quantified by the external standard method. The calibration curve and correlation coefficient was *y* = 1123.86811 *x* + 1837.41231 (*R*^2^ = 0.99211).

### 2.9. Sample Preparation

Fish and chicken samples were purchased from the local market. For the preparation of patties, the muscle tissue was minced through a meat grinder and formed into patties using a circular meat-patties mold (30 g, diameter: 6 cm, height: 1 cm). Chicken patties were fried according to the following conditions of time and temperature: “medium” (5 min, 240 °C), “well-done” (10 min, 240 °C), “very well-done” (15 min, 240 °C), and turning over once a minute in the frying process. The processing time of fish samples was shorted, according to the actual situation, “medium” (3 min, 240 °C), “well-done” (5 min, 240 °C), “very well-done” (10 min, 240 °C), and turning over once a minute in the frying process. After frying, the processed meat samples were minced using a meat grinder and stored in the refrigerator at -20 °C for subsequent use.

The samples for this fluorescence immunoassay were prepared as follows: 4.0 g of the processed meat samples, 8 mL of ethyl acetate, and 2 mL of sodium hydroxide solution (2 mol L^−1^) were added into a 50 mL tube, followed by a vigorous vortex extraction for 5 min. After centrifugation for 5 min (10,000 rpm, 4 °C), the supernatant was collected, and then the precipitate was re-extracted. Next, 10 mL of supernatant was evaporated to dryness with nitrogen gas at 46 °C, and the residue was redissolved in a mixed solution of 4 mL n-hexane and 4 mL methanol. After vortex mixing for 1 min, the lower layer of the above solution was obtained by centrifugation for 5 min (10,000 rpm, 4 °C) and was dried with nitrogen at 46 °C. Finally, the residue was resuspended with 1 mL of the mixed buffer of methanol and PBS buffer (5:95, *V/V*), and the resulting sample solution was used for subsequent analysis. UPLC-MS/MS has been applied to confirm the analysis accuracy of this fluorescence immunoassay.

The samples for UPLC-MS/MS were prepared as follows: 4.0 g of meat samples and 20 mL of extracted buffer (40 g L^−1^ sodium hydroxide, methanol) (70:30, *V/V*) were added into a 50 mL centrifuge tube with a vigorous vortex extraction (5 min). Then, the mixture was centrifuged for 10 min (10,000 rpm, 4 °C). Subsequently, 10 mL of the supernatant was added into a solid-phase extraction column, which had previously been activated with 2 mL of methanol and 3 mL of sodium hydroxide (4 g·L^−1^). Next, the column was washed with 2 mL of n-hexane and 3 mL of mixture solution (4 g·L^−1^ sodium hydroxide and methanol) (45:55, *V/V*) and eluted with 1.5 mL of mixture solution (ethanol and dichloromethane) (10:90, *V/V*). The eluent was dried by nitrogen stream, and the residue was resuspended with 1 mL of mixed buffer (15 mmol L^−1^ ammonium formate buffer, acetonitrile) (50:50, *V/V*). Finally, the solution was filtered by a 0.22 μm microporous membrane before the injection into UPLC-MS/MS.

## 3. Results and Discussions

### 3.1. UCNPs Characterization

**Figure 3:**
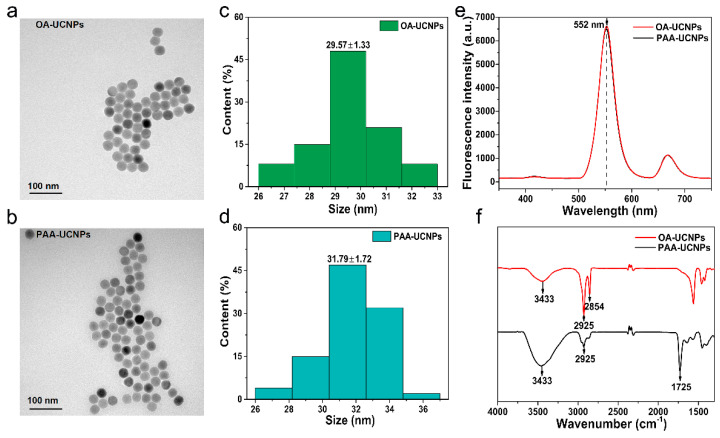
Characterization of the NaYF_4_: Yb, Er UCNPs. (**a**,**b**): Transmission electron microscope (TEM) image of NaYF_4_: Yb, Er UCNPs, (**c**,**d**): Size distribution of NaYF_4_: Yb, Er UCNPs, (**e**): Fluorescence spectrums of NaYF_4_: Yb, Er UCNPs (under 980 nm excitation), (**f**): Fourier transform infrared (FTIR) spectrums of NaYF_4_: Yb, Er UCNPs.

The morphology and size distribution of NaYF_4_: Yb, Er UCNPs were observed by the transmission electron microscope (TEM). The TEM images in Figure 3a,b reveal that UCNPs are spherical in shape, and they have a smooth surface and good monodispersity. Figure 3c,d shows that the average particle size for OA-UCNPs is 29.57 ± 1.33 nm and for PAA-UCNPs is 31.79 ± 1.72 nm. Figure 3e shows that UCNPs have a strong fluorescence emission peak at 552 nm under 980 nm excitation. The Fourier transform infrared spectrums (FTIR) of OA-UCNPs and PAA-UCNPs are compared to judge whether the hydrophilic modification is successful. As displayed in Figure 3f, compared with OA-UCNPs, the spectrum of PAA-UCNPs has a stronger stretching vibration band of the hydroxyl group (around 3433 cm^−1^) and a weaker stretching vibration band of the methylene group of oleic acid (around 2925 cm^−1^ and 2854 cm^−1^). In addition, the spectrum of PAA-UCNPs appears a new characteristic peak around 1725 cm^−1^, which corresponds to the carbonyl stretching vibration of polyacrylic acid. Therefore, the FTIR indicates that ligand exchange can increase the number of carboxyl groups on the surface of OA-UCNPs and the hydrophilic modification is successful.

### 3.2. Optimization of Conditions

Many parameters were involved in the analytic efficiency of the method. We optimized several factors to achieve the optimal analytical performance of this fluorescence immunoassay, including probe preparation and working conditions.

**Figure 4:**
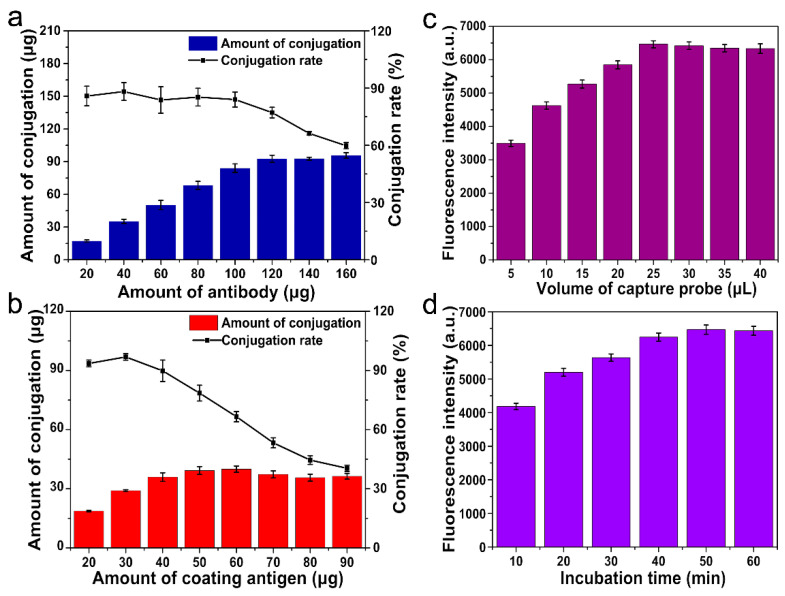
Optimization of the working parameters. (**a**): Optimization of the anti-IQ antibody amount in signal probe, (**b**): Optimization of the coating antigen amount in capture probe, (**c**): Optimization of the added volume of the capture probe in test process with 25 μL of the signal probe, (**d**): Optimization of the incubation time of the signal probe, capture probe and sample solution. Each value is mean of three replicates.

The conjugation amount of the anti-IQ antibody with PAA-UCNPs was investigated. Different amounts (20, 40, 60, 80, 100, 120, 140, and 160 μg) of the anti-IQ antibody were incubated with 100 μL of activated PAA-UCNPs (5 mg·mL^−1^), respectively. Next, the mixture solution was stirred slowly for 3 h at 4 °C. Subsequently, the supernatant of the resulting mixture was separated by centrifugation for 10 min (5000 rpm, 4 °C), and the amount of unreacted anti-IQ antibody in the obtained supernatant was determined by BCA Protein Quantification Kit. The amount of coupled anti-IQ antibody is calculated by subtracting the unreacted anti-IQ antibody in the supernatant from the total amount of added anti-IQ antibody. The conjugation rate is calculated by the formula:

(1) conjugation rate (%)=(mt−m0)/mt×100,

wherein *mt* represents the total amount of added anti-IQ antibody, and *m0* means the amount of unreacted anti-IQ antibody. As displayed in Figure 4a, the coupling amount increases with the anti-IQ antibody addition amount increasing from 20 μg to 120 μg. As the amount of added anti-IQ antibody is greater than 120 μg, no noticeable change in the coupling amount is observed, and the coupling efficiency gradually decreases. Finally, 120 μg of the anti-IQ antibody with 77.16% of conjugation rate is selected as the optimal addition amount of anti-IQ antibody to preparing the signal probe.

The conjugation amount of the coating antigen with MNPs was investigated too. Different amounts (20, 30, 40, 50, 60, 70, 80, and 90 μg) of the coating antigen were incubated with 100 μL of activated MNPs (5 mg·mL^−1^), respectively. Then, the mixture solution was reacted at room temperature on the horizontal reciprocating shaker for 3 h. After being isolated by a magnet, the content of coating antigen in the supernatant was determined. The amount of coupled coating antigen was calculated by subtracting the unreacted coating antigen in the supernatant from the total amount of added coating antigen. The conjugation rate was calculated by the formula:

(2) conjugation rate (%)=(mt−m0)/mt×100.

Herein, *mt* represents the total amount of added coating antigen, and *m0* means the amount of unconnected coating antigen. As displayed in Figure 4b, the coupling amount increased with the added quantity of coating antigen from 20 μg to 50 μg. As the addition quantity of coating antigen is greater than 50 μg, no noticeable increase in the coupling amount is observed, and the coupling rate decreases. Therefore, 50 μg of the coating antigen with 78.53% of conjugation rate is selected as the optimal addition amount of coating antigen to preparing the capture probe.

We also assessed the effect of the addition amount of capture probe on the fluorescence intensity of the immunoassay. Various amounts (5, 10, 15, 20, 25, 30, 35, and 40 μL) of the capture probe (2.5 mg mL^−1^) were incubated for 1 h with 25 μL of signal probe (2.5 mg mL^−1^), respectively. Then immune complex was separated by a magnet and washed three times with PBS buffer, and the fluorescence intensity was recorded by the fluorescence spectrophotometer. As displayed in Figure 4c, a gradual increase in fluorescence intensity is observed with the increasing addition of the capture probe. When the added volume of the capture probe is equal to 25 μL, the fluorescence intensity reaches saturation, so 25 μL of the capture probe is used as the optimal addition to establishing the analytical method. The effect of the incubation time was also investigated. As shown in Figure 4d, the fluorescence intensity of the immune complex gradually increases with the extension of incubation time, and the saturated signal is observed at 50 min and then remains stable. Thus, 50 min of incubation time was selected in this method to ensure adequate immune response and save test time.

### 3.3. Analytical Performance

**Figure 5:**
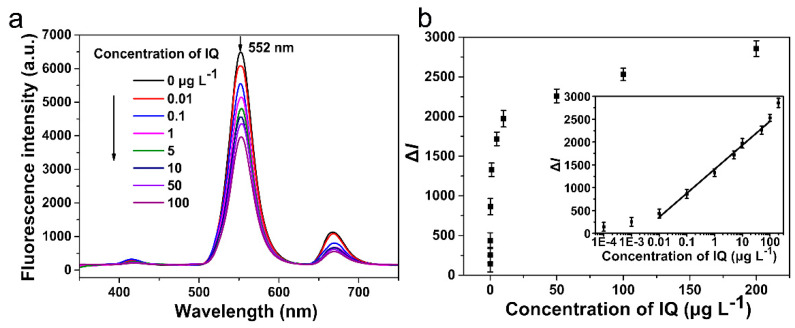
Detection of 2-amino-3-methylimidazo [4,5-f] quinoline (IQ) with fluorescence immunoassay. (**a**): Fluorescence intensity of the immune complexes with varying concentrations of IQ, (**b**): Standard curve of the fluorescence immunoassay for IQ in the PBS buffer. Each value is mean of three replicates.

According to the optimized conditions, a fluorescence immunoassay was established. Various concentrations (0, 0.0001, 0.001, 0.01, 0.1, 1, 5, 10, 50, 100, and 200 μg·L^−1^) of IQ standard solution were detected by this fluorescence immunoassay, and the emission peak at 552 nm was selected as a signal peak. As shown in Figure 5a, the maximum fluorescence intensity at 552 nm is obtained when the concentration of IQ is 0 μg L^−1^, and the fluorescence intensity gradually decreases with the increasing concentration of IQ. The reduces fluorescence intensity Δ*I*, which is calculated by the formula:

(3) $$\Delta I=I_{0}-I$$

wherein *I*_0_ and *I* represent the fluorescence intensity at 552 nm in the absence and presence of IQ, respectively. There exists a proportional relationship between Δ*I* and the concentration of IQ. As displayed in Figure 5b, the fluorescence intensity exhibits good linearity with the IQ concentration range of 0.01 to 100 μg L^−1^, with a linear equation of y = (524.869 ± 20.586) lg(x) + (1405.077 ± 28.949) (R^2^ = 0.991), which y represents the reduces fluorescence intensity, and x means the concentration of IQ. According to the 3σ principle, the detection limit is 0.007 μg·L^−1^ for this fluorescence immunoassay. The half-maximum inhibition concentration (IC_50_) and the limit detection (IC_15_) of the traditional ELISA using same anti-IQ antibody and coating antigen are 52.67 μg·L^−1^ and 1.12 μg·L^−1^, respectively. In contrast, the fluorescence immunoassay based on the signal amplification capability of UCNPs and the rapid separation ability of MNPs has higher sensitivity and shorter analysis time for IQ detection.

### 3.4. Specificity Assessment

**Figure 6:**
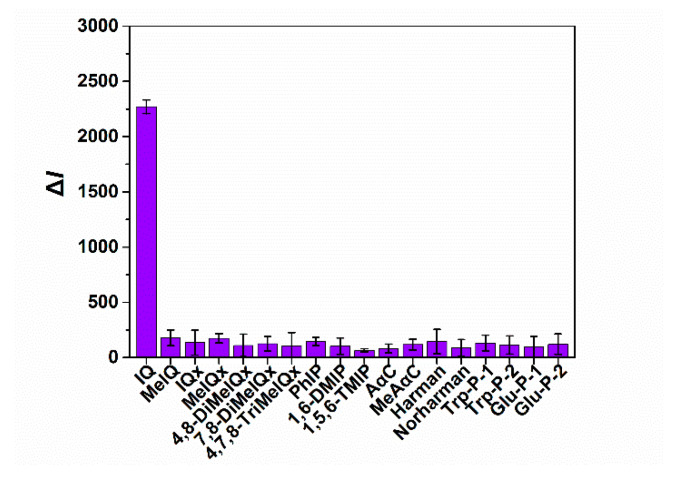
Specificity analysis of the fluorescence immunoassay. Each value is mean of three replicates. (2-amino-3-methylimidazo [4,5-f] quinoline, (IQ); 2-amino-3,4-dimethylimidazo [4,5-f] quinoline (MeIQ); 2-amino-3-methylimidazo [4,5-f] quinoxaline (IQx); 2-amino-3,8-dimethylimidazo [4,5-f] quinoxaline (MeIQx); 2-amino-3,4,8-trimethylimidazo [4,5-f] quinoxaline (4,8-DiMeIQx); 2-amino-3,7,8-trimethylimidazo [4,5-f] quinoxaline (7,8-DiMeIQx); 2-amino-3,4,7,8-tetraimethylimidazo [4,5-f] quinoxaline (4,7,8-TriMeIQx); 2-amino-1-methyl-6-phenylimidazo [4,5-b] pyridine (PhIP); 2-amino-1,6-dimethylimidazo [4,5-b] pyridine (1,6-DMIP); 2-amino-1,5,6-trimethylimidazo [4,5-b] pyridine (1,5,6-TMIP); 2-amino-9H-pyrido [2,3-b] indole (AαC); 2-amino-3-methyl-9H-pyrido [2,3-b] indole (MeAαC); 1-methyl-9H-pyrido [3,4-b] indole (Harman); 9H-pyrido [3,4-b] indole (Norharman); 3-amino-1,4-dimethyl-5H-pyrido [4,3-b] indole (Trp-P-1); 3-amino-1-methyl-5H-pyrido [4,3-b] indole (Trp-P-2); 2-amino-6-methyldipyrido [1,2-a:3′,2′-d] imidazole (Glu-P-1), 2-amino-dipyrido [1,2-a:3′,2′-d] imidazole (Glu-P-2)).

To estimate the specificity of this method, other seventeen HAAs (including MeIQ, IQx, MeIQx, 4,8-DiMeIQx, 7,8-DiMeIQx, 4,7,8-TriMeIQx, PhIP, 1,6-DMIP, 1,5,6-TMIP, AαC, MeAαC, Harman, Norharman, Trp-P-1, Trp-P-2, Glu-P-1, Glu-P-2), at 50 μg L^−1^ were tested by the fluorescence immunoassay. Compared with IQ, the decrease fluorescence intensity (ΔI) induced by the other seventeen HAAs is negligible (Figure 6). Therefore, the fluorescence immunoassay based on the anti-IQ antibody could be a specific tool for the detection of IQ.

### 3.5. Sample Analysis

To assess the practicability of this fluorescence immunoassay, the IQ content in processed meat samples was analyzed by the proposed method. As shown in Table 1, the IQ content detected by the fluorescence immunoassay in pan-fried chicken and fish samples varies from 2.11 to 3.47 μg·kg^−1^ and 1.35 to 2.85 μg·kg^−1^, respectively. It could be noted that the amount of IQ was affected by the fry time. During the setting time, as the fry time was prolonged, the IQ content increased gradually although the increase was not significant after further increasing fry time (*p* < 0.05). This may be due to the rapid evaporation of water on the surface of the pan-fried patties during heating, forming a hard crust, which prevents the internal precursor from being transferred out. In conclusion, the amount of IQ in pan-fried patties shows variation depending on the effect of doneness degree and type of meat. The IQ content in the pan-fried chicken samples was similar to the previous study [32]. UPLC-MS/MS was used to verify the feasibility and accuracy of this method. The test result shows no noticeable differences between them, indicating that the fluorescence immunoassay can be used for the determination of IQ in real heat processed meat.

## 4. Conclusions

In this work, we have established a novel fluorescence immunoassay for the detection of IQ in heat-processed meat. Through the signal amplification effect of UCNPs, this fluorescence immunoassay has a low detection limit at 0.007 μg·L^−1^, which is significantly lower than the detection limit of traditional ELISA using the same anti-IQ antibody and coating antigen. The fluorescence immunoassay has a good specificity to IQ and no cross-reactivity with other HAAs. This fluorescence immunoassay has been successfully used to detect the IQ content in pan-fried chicken and fish patties with different frying time, and the results are consistent with the UPLC-MS/MS analysis. Thus, the fluorescence immunoassay could be a rapid, specific, and sensitive tool to monitor the strong mutagenic and probable carcinogenic IQ in processed foods.

## Figures and Tables

**Table 1:** Comparison of this method with UPLC-MS/MS for the analysis of 2-amino-3-methylimidazo [4,5-f] quinoline (IQ) in pan-fried meat patties.

Sample	Fry Time (min)	This Method (μg kg^−1^)		UPLC-MS/MS (μg kg^−1^)	
		(Mean ± SD)	CV (%)	(Mean ± SD)	CV (%)
Chicken	0	ND	-	ND	-
	5	2.11 ± 0.27 ^b^	12.80	1.95 ± 0.23 ^b^	11.79
	10	2.56 ± 0.37 ^b^	14.45	2.44 ± 0.30 ^ab^	12.30
	15	3.47 ± 0.44 ^a^	12.68	3.15 ± 0.54 ^a^	17.14
Fish	0	ND	-	ND	-
	3	1.35 ± 0.18 ^b^	13.33	1.19 ± 0.09 ^b^	7.56
	5	2.56 ± 0.21 ^a^	8.20	2.47 ± 0.15 ^a^	6.07
	10	2.85 ± 0.40 ^a^	14.04	2.82 ± 0.32 ^a^	11.35

Mean: Each result is the average of three determinations; SD: Standard deviation; CV: Coefficient of variation; ND: Not detected; ^a,b^: Means with different letters in the same sample type and analysis method are significantly different due to the effect of fry time (*p* < 0.05). The differences between variables were tested for significance using ANOVA and Duncan’s multiple range test. Differences between means were considered significantly different at *p* < 0.05 (SPSS for Window 24.0).

## Data Availability

Not applicable.
